# Multilevel Screening Platform Utilizing Cellular and Zebrafish Models to Identify Short Peptides with High Improvement of Motor Neuron Growth

**DOI:** 10.3390/ijms27010281

**Published:** 2025-12-26

**Authors:** Bing-Chang Lee, Chun-Cheng Wang, Shan-Pin Chen, Huai-Jen Tsai

**Affiliations:** 1 Department of Life Science, Fu Jen Catholic University, New Taipei City 242, Taiwan; bingchanglee@ntu.edu.tw; 2 Institute of Molecular and Cellular Biology, National Taiwan University, Taipei 106, Taiwan; r12b43006@ntu.edu.tw (C.-C.W.); r13b43024@ntu.edu.tw (S.-P.C.)

**Keywords:** motor neurons, neuroprotection, short peptides, phenotypes, zebrafish

## Abstract

Zebrafish is emerging as a model animal for phenotype-based drug screening. Drugs screened from the zebrafish platform have advanced into clinical trials, underscoring their translational potential. Amyotrophic lateral sclerosis is a progressive motor neurons (MN) degenerative disease with few approved drugs. Previously, supplementation with exogenous recombinant phosphoglycerate kinase 1 (Pgk1) was found to improve MN growth through its interaction with receptor Eno2. To bypass the high complexity and cost of full-length Pgk1 production, a short segment within Pgk1 (M08) was predicted as the key motif interacting with Eno2, and a zebrafish phenotypic screening platform was established to find the most neurotrophic compound(s) among M08 and its mutants. We first found that M08-injected zebrafish embryos significantly increased branched caudal primary MNs (CaPMNs). However, compared to M08 (59.20 ± 1.80%), M039, among 17 mutants further screened, showed even more improvement of branched CaPMNs, up to 74.54 ± 3.73%. Next, when we administered the M039 peptide to *C9ORF72*-knockdown ALS-like zebrafish embryos, it improved axonal growth and swimming ability. Then, we employed a cellular model as a secondary screen, and M039 exhibited improved neurite outgrowth of MN (NOMN) and reduced p-Cofilin in NSC34 neural cells grown in ALS-like condition. Therefore, by using a zebrafish MN phenotype as a primary screening platform, we identified a mutated short peptide M039 having the most pronounced positive effect on improving neurite growth among all 17 mutants in comparison to parental M08, demonstrating the feasibility of zebrafish screening as a cost-effective strategy for finding promising neuroprotective short peptides that serve as neurotherapeutic potentials.

## 1. Introduction

As a powerful vertebrate model in biomedical research, the zebrafish (*Danio rerio*) possesses large and abundant eggs, high fecundity, light-inducible spawning, transparent embryos, rapid development and easy genetic manipulation [1]. The zebrafish genome shares about 70% similarity with the human genome, and roughly 84% of human disease-related genes correspond with homologs in zebrafish [2]. With these characteristics, the zebrafish is well suited as an animal model to perform preliminary drug screening and toxicity evaluation with the aim of bridging basic science and clinical translation [3,4].

Over the past three decades, most drug development efforts have relied on a target-based approach whereby a disease-associated molecular target is first chosen, potential drugs are designed and developed, and a lead compound is finally tested in preclinical and clinical trials. However, more recent approaches discover first-in-class drugs through phenotype-based screening. Swinney and Anthony [5] analyzed new molecular entities approved by the US FDA from 1999 to 2008 and found that 62% had been identified by phenotype-based screening in comparison to only 38% through target-based discovery. The phenotype-driven strategy allows researchers to observe the overall effects of compounds on cells and/or organisms without predefining specific molecular targets. Given its ability to display morphological, behavioral, physiological, and molecular phenotypes simultaneously, the zebrafish can serve as an ideal whole-organism model for phenotype-based drug discovery [6,7]. While zebrafish disease model currently represents only a small portion of phenotypic screening platform, their use is rapidly expanding, especially in the fields of central nervous system disorders and congenital developmental diseases where zebrafish show high sensitivity and predictive value [8].

Thus far, zebrafish model has been instrumental in advancing therapeutic agents to clinical trials, highlighting their translational potential in early-stage drug development. For example, ORC-13661, an oral medicine developed by Oricula Therapeutics to protect against hearing loss, was initially screened from zebrafish lateral-line hair cells. Currently, it has progressed to Phase II clinical trials [9]. Clemizole, a histamine H 1 receptor antagonist of the benzimidazole group, was identified as an anti-seizure compound in a zebrafish model of Dravet syndrome, and it showed efficacy in reducing seizure frequency in patients. It has progressed to Phase III trial with continued use, and follow up is permitted after successfully completing the trial [10,11]. The success of these drugs in clinical trials highlights the application of the zebrafish model in phenotype-based screening, especially for complex, polygenic, and neurodegenerative diseases [4,9].

In the context of neurological disorders, zebrafish have highly conserved central and peripheral nervous systems, making them extremely useful in studies of neurodevelopment, synaptic function, regeneration, and neurodegeneration. Their transparent bodies and visible axonal structures make them particularly suitable for investigating motor neurons (MN) disorders. Amyotrophic lateral sclerosis (ALS), for instance, is a progressive neurodegenerative disease affecting both upper and lower MN, and most patients die from respiratory failure within 3 to 5 years of symptom onset [12,13]. Although mutations in genes, such as *SOD1*, *TARDBP*, *FUS*, and *C9ORF72*, have been linked to ALS through mechanisms that include protein misfolding, oxidative stress, and neuroinflammation, the etiology of approximately 80% of ALS cases remains unknown [14,15,16]. Currently, approved drugs, such as Riluzole and Edaravone, may help in slowing disease progression, but their effects on improving motor function and prolonging survival are limited [17], underscoring the urgent need for the development of new neuroprotective agents with multimodal mechanisms.

Previous studies have shown that the axonal growth inhibitor NogoA is abnormally elevated in ALS patients and mouse model [18,19] and that its overexpression in skeletal muscle cells suppresses motor neuron development. Overexpression of NogoA in muscle cells also suppresses the release of factors beneficial for motor neuron growth, including Phosphoglycerate kinase 1 (Pgk1). However, supplementation with exogenous recombinant Pgk1 was shown to partially rescue the inhibition of neurite outgrowth of motor neurons (NOMN) and, hence, improve locomotive capability [20]. Therefore, apart from its canonical role in glycolysis, Pgk1 can also act as an extracellular modulator (ePgk1), interacting with the 404th–431st amino acid (aa) segment of the neural membrane receptor Enolase 2 (Eno2) through its own 325th–417th aa segment to increase NOMN in length (in vitro) and promote branched caudal primary MNs (CaPMNs) in number (in vivo) [21,22]. However, from a pharmacological viewpoint, large-scale production of full-length Pgk1 with a molecular weight of ~45kDa would be challenging by the cost and complexity.

To address this challenge, we know that protein-protein interface-based peptide design has been widely used in developing new biotherapeutics, showing improved binding affinity and in vivo stability [23]. Accordingly, based on computational structural analysis, a 16-aa segment (residues from 345th to 360th) within Pgk1 was predicted as a potential critical motif for Eno2 binding [21]. Therefore, we asked whether (1) this truncated short peptide, named M08, might increase both NOMN and CaPMNs; and (2) its mutated peptides might show additional, or heightened, neuroprotective properties. For answer to these questions, we first employed M08 as a template and generated 17 mutations, including site-directed single and multiple aa mutations through both conservative and non-conservative aa substitutions. Next, we employed a multilevel platform consisting of both cellular (in vitro) and zebrafish (in vivo) models to screen putative short mutated peptide(s) that demonstrated the best overall neuroprotective properties in comparison to parental M08. In the cellular system, we measured the length of NOMN derived from NSC34 neural cells, and we quantified p-Cofilin expression level, a growth cone collapse marker [24], because reduced p-Cofilin leads to increased active cofilin which, in turn, promotes NOMN. For the in vivo system, we used transgenic zebrafish line *Tg(mnx1:GFP)* expressing GFP specifically in motor neurons. The fluorescence intensity allows us to easily count the number of branched CaPMNs and observe the axonal shortage of ALS-like phenotype in *C9ORF72*-knockdown embryos. Therefore, this multilevel screening platform allows the analysis of the 17 mutated peptides by functional outcomes, namely axonal extension and swimming activity, offering, in turn, potential mutant short peptide(s) with the properties most likely to achieve strong MN improvement.

## 2. Results

### 2.1. Effects of Supplementary Pgk1-Derived Short Peptide and Its Mutants on the Formation of Branched CaPMNs in Zebrafish Embryos

Short peptide M08 is a truncated form originated from the 345th to 360th segment of full-length Pgk1. It has been reported as a critical motif involved in the promotion of axonal growth through the interaction between ligand ePgk1 and receptor Eno2 [21,22]. In this study, we used M08 as a template and performed mutation to generate 17 M08-based mutants (Table 1), including single-aa, double-aa, and more than three-aa mutations, as well as one five-aa-addition and one four-aa-deletion. The changes in charge and polarity of each mutant compared to the same properties of benchmark M08 are listed in Table 1.

**Figure 1:**
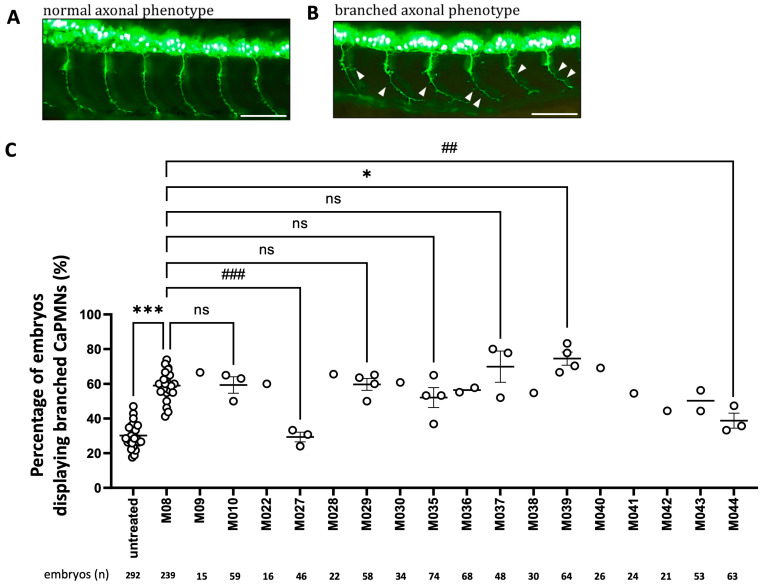
Effect of short peptide and its mutants on the development of branching caudal primary motor neurons (CaPMNs) in zebrafish embryos. Using ICV injection, we delivered Pgk1-derived short peptide and its mutants individually into transgenic zebrafish *Tg(mnx1:GFP)* embryos at the 20–22 hpf stage. The occurrence rate (in percentage) of branched CapMN within 11th to 16th somites was calculated. (**A**) CaPMN in untreated embryos and (**B**) branched CaPMN phenotype indicated by white arrows were observed under fluorescence stereo microscopy. Scale bar = 50 μm (**C**) Quantification of the percentage of embryos displaying branched CaPMN-examined embryos (*n*; listed on the bottom row) injected with various short peptides as indicated. The untreated group served as a control. Each experiment was performed with at least 15 embryos, and its average datum was represented as a circle dot. Some experimental groups were performed with at least three independent experiments. Their data were averaged from all experiments and presented as mean ± SEM. Horizontal bars indicated the mean value of each group. Statistical analysis, if any, was assessed using one-way ANOVA, followed by Tukey’s multiple comparison test (ns, not significant; increased significance at *, *p* < 0.05; ***, *p* < 0.001, while decreased significance at ##, *p* < 0.01; ###, *p* < 0.001).

CaPMNs are located at the myoseptum and primarily innervate ventral trunk musculature [25]. Previously, Lin et al. [20] demonstrated that muscle-specific overexpression of Pgk1 in embryos promotes the formation of branched CaPMNs. In this study, we performed an intracerebroventricular (ICV) injection of truncated peptide M08 (9.2 ng/embryo) into zebrafish *Tg(mnx1:GFP)* embryos at 20–22 hpf. Afterwards, we counted the number of embryos displaying the formation of branched CaPMNs between the 11th and 16th somites in 30-hpf embryos. Results demonstrated that the untreated group exhibited an average branched CaPMNs ratio of 31.88 ± 1.51% (*n* = 502 embryos), while M08 peptide administration significantly increased branched CaPMNs to 59.20 ± 1.80% (*n* = 449 embryos) (Figure 1). Thus, short peptide M08 can promote the formation of branched CaPMNs.

We then used a zebrafish phenotypic screening platform to evaluate the effect of various mutated peptides on the formation of branched CaPMNs. As before, we employed ICV injection to deliver different mutant peptides individually into 20–22 hpf embryos, followed by an analysis of the growth patterns of six branched CaPMNs. Compared to the increase in branched CaPMNs of M08 (59.20 ± 1.80%), three distinct levels of increased branched CaPMNs among the 17 mutants could be first categorized as superior to that of M08, including M09 (66.66%), M028 (65.62%), M037 (69.92 ± 8.99%), M039 (74.54 ± 3.73%), and M040 (69.23%), suggesting that these mutated aa residues may favor neurite growth-promoting activity by their improved charge, polarity or structural conformation. Among these, mutant M039 showed the most improvement in the formation of branched CaPMNs among 17 mutants and wild-type M08 (Figure 1).

A second class of mutants showed an increase in CaPMNs formation equal to that of M08, including M010 (59.38 ± 4.72%), M022 (60%), M029 (59.72 ± 3.42%), M030 (60.86%), M035 (52.13 ± 5.79%), M036 (56.47 ± 1.30%), M038 (54.83%), M041 (54.50%) and M043 (50.33 ± 5.93%), suggesting that these aa substitutions did not markedly alter the intrinsic branching-promotion potency of M08. Finally, a third class of mutation showed an increase in CaPMNs inferior to that of M08, including M027 (29.36 ± 2.78%), M042 (44.44%) and M044 (38.81 ± 4.35%), suggesting that these mutated aa(s) might be key aa residues responsible for biological activity because their substitutions led to the reduced ability to promote branched CaPMNs.

Overall, the use of the zebrafish phenotypic screening platform allowed us to quickly identify mutant M039 as having the most pronounced positive effect on improving the formation of branched CaPMNs as high as 74.54 ± 3.73%, significantly higher than that of M08 (59.20 ± 1.80%).

### 2.2. Administration of Mutant Short Peptide M039 Resulted in the Partial Rescue of Shortened Axonal Growth Defect in ALS-like Zebrafish Embryos

**Figure 2:**
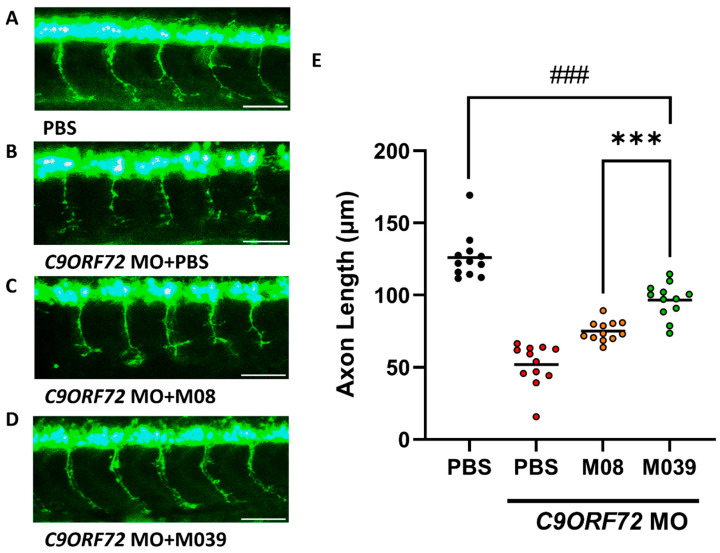
The ALS-like phenotype that occurred in *C9ORF72*-knockdown zebrafish embryos could be best rescued by injection of mutant peptide M039. (**A**–**D**) Representative images of axonal growth of motor neurons (MN) observed in 11th to 15th somites of MN-GFP-tagged *Tg(mnx1:GFP)* zebrafish embryos at 30 hpf under fluorescence stereo microscopy. (**A**) Non-MO-injected group. Injection of PBS into the brain ventricle at 20–22 hpf served as a non-MO-injected control group. (**B**–**D**) MO-injected groups. Embryos were injected with *C9ORF72*-MO at the one-cell stage, followed by a rescue experiment through injection of (**B**) PBS, which served as a rescue control, (**C**) short peptide M08, or (**D**) mutant short peptide M039 at 20–22 hpf. Scale bar = 50 μm (**E**) Quantification of the averaged axonal length of CaPMNs in each group, as indicated, at 30 hpf. Axonal length (μm) of a single CaPMN, represented as a small circle, was averaged from measuring five continuous CaPMNs in each embryo. In each experiment, four embryos were carried out for each group. Three independent experiments were performed. In total, 12 embryos were studied per group; each group was marked with a different color, and horizontal bars indicated the mean value of each group. Statistical analysis was performed using one-way ANOVA, followed by Tukey’s multiple comparison test (***, *p* < 0.001; ###, *p* < 0.001).

As stood out by the primary screening described above, the branched CaPMNs improvement of M039 was higher than that of M08. Next, we went further to know whether the therapeutic potential of M039 is also better than that of M08 in terms of improving MN function in an ALS disease model. In ALS pathology, *C9ORF72* abnormality impairs neural development, leading to the suppression of axonal growth, which, in turn, causes markedly shortened axons [26]. Thus, we generated a transient zebrafish ALS-like model using antisense morpholino oligonucleotides (MO) for *C9ORF72* mRNA to specifically knock down *C9ORF72*, followed by an analysis of the effects on axonal growth. In detail, one nanogram of *C9ORF72*-MO was microinjected into *Tg(mnx1:GFP)* zebrafish embryos at one-cell stage, followed by ICV injection of phosphate-buffered saline (PBS), M08 or M039 peptides in the amount of 9.2 ng per embryo at 20–22 hpf. At 30 hpf, axonal growth was measured. Results showed that the average axonal length was 122.20 ± 2.76 μm in the control embryos injected with PBS instead of MO. A statistical comparison between PBS (non-MO-injected control) and M039 showed a significant decrease in the difference, indicating that the axons could not be restored to their original length but did exhibit a partial rescue effect (Figure 2A,E). However, the axonal length was significantly reduced to 51.47 ± 4.94 μm in the *C9ORF72*-MO-injected embryos, even though rescued by PBS injection (Figure 2B,E), suggesting that *C9ORF72* deficiency led to the strong suppression of axonal growth extension, resulting in an ALS-like phenotype (Figure 2B). In complete contrast, axonal length was partially rescued to 74.68 ± 2.37 and 95.75 ± 3.89 μm in the *C9ORF72*-MO-injected embryos administered with short peptide M08 (Figure 2C,E) and M039 (Figure 2D,E), respectively. This line of evidence suggested that supplementary administration of both M08 and M039 could effectively mitigate axonal shortening in *C9ORF72*-MO-induced ALS-like zebrafish embryos. Moreover, M039 exhibited more significant partial rescue of impaired MN compared to that of M08 (Figure 2E).

### 2.3. Administration of Mutant Short Peptide M039 Markedly Improved Swimming Defect in ALS-like Zebrafish Embryos

**Figure 3:**
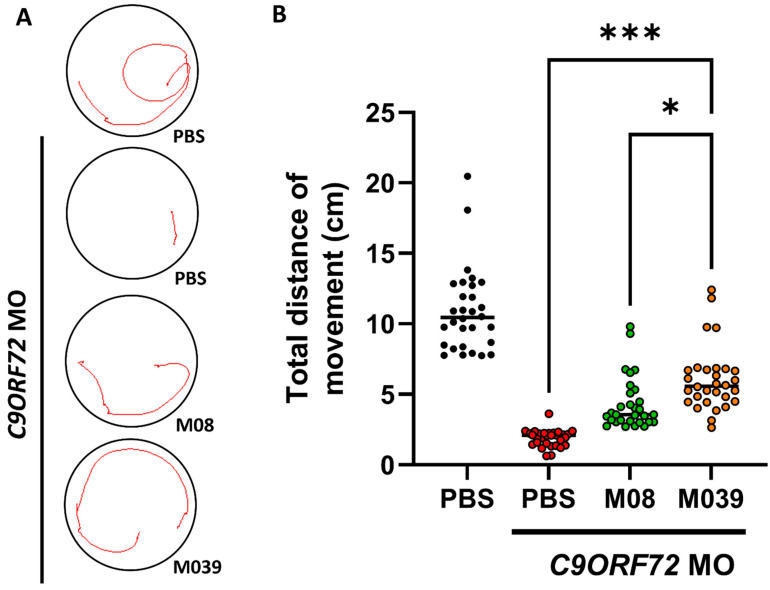
The swimming defect that occurred in ALS-like *C9ORF72*-knockdown zebrafish embryos was significantly rescued by injection of mutant short peptide M039. (**A**) Embryos from the zebrafish transgenic line *Tg(mnx1:GFP)* injected with PBS served as a non-MO-injection control, while embryos injected with *C9ORF72*-MO at one-cell stage, followed by ICV injection with PBS (MO-injection control), M08 or M039 peptide, as indicated, at 20–22 hpf, served as MO-treated groups. The locomotive trajectory of every larva that responded to three consecutive tail touch-escape movements was recorded. (**B**) Total swimming distance (cm) of every larva was calculated by averaging three times and presented as one small circle. For each experiment, ten larvae were used for each group. Data were collected from three independent lots of embryos. In total, 30 embryos were studied per group, each group was marked with a different color, and the mean value of each group was presented as a horizontal bar. Statistical analysis was performed using one-way ANOVA, followed by Tukey’s multiple comparison test (*, *p* < 0.05; ***, *p* < 0.001).

Previous studies have reported that zebrafish embryos treated with *C9ORF72*-MO showed behavior that reflected impaired MN, i.e., reduced swimming ability in larvae owing to shortened axonal length [26]. However, we aimed to compare the rescue of this behavior between M08 and M039 in an ALS disease model in terms of MN function. Therefore, we employed *C9ORF72*-MO-induced ALS-like zebrafish to assess short-distance swimming behavior. Zebrafish embryos derived from the zebrafish transgenic line *Tg(mnx1:GFP)* were injected with *C9ORF72*-MO to knock down *C9ORF72*, followed by ICV injection of the peptide. The tail touch-evoked escape response was performed. In the PBS-treated control group, the average swimming distance was 10.92 ± 0.54 cm (Figure 3A), while embryos injected with *C9ORF72*-MO and treated with PBS via ICV exhibited a significant reduction in swimming distance to 1.89 ± 0.11 cm (Figure 3B), suggesting that severe impairment of MN function had occurred in *C9ORF72*-knockdown embryos. However, *C9ORF72*-MO-treated embryos administered with ICV injection of Pgk1-derived truncated peptides M08 and M039 displayed significant recovery of swimming distance to 4.32 ± 0.34 and 6.05 ± 0.42 cm, respectively (Figure 3B). Collectively, this finding demonstrated that supplementation of either M08 or M039 peptide could effectively alleviate motor loss-of-function caused by *C9ORF72* knockdown and that mutant peptide M039 exceeded the performance of M08 in rescue efficacy. Statistical analysis using one-way ANOVA followed by Tukey’s post hoc test further revealed that the swimming distance of *C9ORF72*-MO embryos treated with M039 was significantly higher than that of the MO + PBS group, confirming a robust rescue effect by the mutant peptide (Figure 3B).

### 2.4. Exogenous Addition of Mutant M039 Could More Effectively Promote the Length of NOMN Compared to Benchmark M08

**Figure 4:**
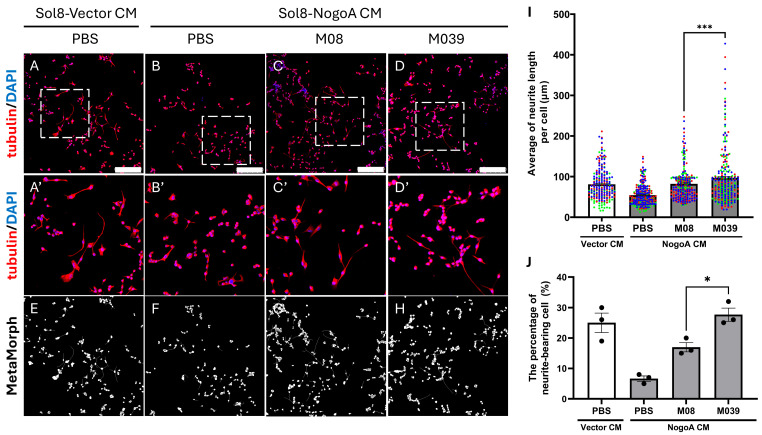
Neurite outgrowth of motor neurons (NOMN) was significantly increased in neural cells cultured in ALS-like conditions supplemented with mutant peptide M039. (**A**–**D**) Confocal microscopy images showing NOMN derived from NSC34 neural cells cultured in the following CM obtained from culturing: (1) cells harboring Sol8-Vector (Vector CM) and treated with PBS (control group); (2) cells harboring Sol8-NogoA (NogoA CM) and treated with PBS (control group of ALS-like condition); (3) cells harboring Sol8-NogoA and treated with peptide M08 (M08); and (4) treated with peptide M039 (M039). Nuclei were stained with DAPI (blue), and microtubules were detected by an anti-tubulin antibody labeled in red. Scale bar = 250 μm. (**A’**–**D’**) Magnified views of the box areas marked with dashed lines in the corresponding panels of (**A**–**D**). (**E**–**H**) Quantification of neurite length using the Neurite Outgrowth module in MetaMorph 7.8.0 software. Cell bodies and neurites are marked in white. (**I**) Statistical analysis. For each trial, 100 cells harboring relatively longer neurites were chosen and their neurite lengths (μm) were measured and presented as a dot on the scatter plot. The averaged neurite length (μm) per cell was calculated. Each experimental group was performed three independent trials and each trial was marked by one color. The total mean value of each group was averaged from three independent trials and presented as a horizontal bar. (**J**) The percentage of neurite-bearing cells (neurite length > 100 μm) among 100 cells in each trial was counted. Data were averaged from three independent experiments and presented as mean ± SEM. Statistical significance was determined by one-way ANOVA, followed by Tukey’s multiple comparison test (*, *p* < 0.05; ***, *p* < 0.001).

Next, we switched to use NSC34 neural cells to examine the efficacy of short peptide M039 in promoting NOMN compared to that of M08. To accomplish this, NSC34 cells were cultured in 1.5% FBS serum-deprived medium (SDM) for 48 h, followed by incubation with Sol8-NogoA conditioned medium (CM) to suppress NOMN in the presence or absence of M08 or M039 peptide. After 4 days of treatment, we observed cell morphology and found that the percentage of long-axon-bearing cells and total neurite length were 25 ± 3.22% and 81.08 ± 2.12 μm, respectively, in the control group, consisting of NSC34 neural cells incubated in the CM from cultured Sol8 cells harboring the empty cloning vector in the presence of PBS (Vector CM + PBS) (Figure 4A,I,J). In contrast, the percentage of long-axon-bearing cells and total neurite length were significantly reduced to 6.67 ± 0.88% and 53.95 ± 1.43 μm, respectively, in the Sol8-NogoA CM + PBS group (Figure 4B,I,J), suggesting that the NOMN of NSC34 cells incubated in Sol8-NogoA CM was inhibited. However, the percentage of long-axon-bearing cells and NOMN length were increased to 17 ± 1.53% and 82.25 ± 2.33 μm, respectively, in cells incubated with Sol8-NogoA CM and short peptide M08 (Figure 4C,I,J). Meanwhile, when NSC34 cells were incubated with Sol8-NogoA CM and mutant peptide M039, these two parameters were remarkably increased to 27.67 ± 2.19% and 96.96 ± 3.87 μm, respectively (Figure 4C,D,I,J). Thus, while both M08 and M039 could increase NOMN length, evidence shows that M039 could more effectively promote NOMN compared to M08 under in vitro conditions.

### 2.5. Exogenous Addition of Mutant Short Peptide M039 Displayed a Greater Efficacy to Reduce p-Cofilin Level in ALS-like Cells

**Figure 5:**
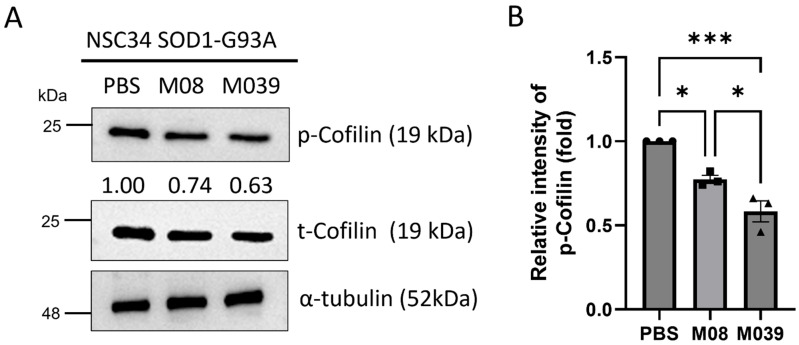
The protein level of p-Cofilin was significantly reduced in ALS-like neural cells treated with mutant short peptide M039. (**A**) Western blot analysis. The protein levels of p-Cofilin (growth cone collapse marker) and total Cofilin (t-Cofilin) expressed in SOD1-G93A NSC34 neural cells treated with PBS (control), peptide M08, or M039, as indicated, were presented. The protein level of α-tubulin served as an internal loading control. The relative intensity of the p-Cofilin band (in fold change) of each group compared to that of the PBS control is listed under each lane. (**B**) Quantification of the expression level of p-Cofilin. The relative intensity of p-Cofilin against α-tubulin within each group was calculated and represented by the fold change in p-Cofilin over that obtained from the PBS control group, normalized to 1. Data were averaged from three independent trials and presented as mean ± SEM. Statistical analysis was performed using one-way ANOVA, followed by Tukey’s multiple comparison test (*, *p* < 0.05; ***, *p* < 0.001).

It has been reported that extracellular addition of recombinant Pgk1 in medium could reduce the protein level of p-Cofilin-S3, a growth cone collapse marker, thereby favoring tubulin formation to promote the NOMN of NSC34 cells [20]. Therefore, we asked if administration of either M08 or M039 in the CM containing from culturing SOD1-G93A NSC34 cells could sufficiently decrease p-Cofilin levels to promote MN differentiation in these ALS-like cells. After treatment, total proteins were extracted for Western blot analysis. Compared with the PBS control group set as 1, the relative expression of p-Cofilin was significantly decreased to 0.77 ± 0.02 and 0.58 ± 0.06 (*n* = 3) in the M08 and M039 groups, respectively (Figure 5A,B), suggesting that administering M08 and M039 could cause a significant reduction in p-Cofilin levels in ALS-like cells compared with the control. Nevertheless, compared to M08, M039 displayed a greater efficacy to suppress Cofilin phosphorylation in an ALS-like environment and, hence, a greater ability to promote the differentiation of MN.

## 3. Discussion

### 3.1. Zebrafish Phenotypic Screening Platform Demonstrates That Pgk1-Derived Original Short Peptide and Its Mutants Show Functional Disparity in Promoting Branched Formation of CaPMNs

We used zebrafish as our in vivo screening model because of the transparency of embryos and the availability of a transgenic line, allowing direct observation of MN development and morphogenesis. The *Tg(mnx1:GFP)* line with GFP-labeled MN further enabled real-time visualization of MN development and morphogenesis in living organisms. Here, we employed the zebrafish phenotypic platform and demonstrated that a truncated segment of Pgk1, the wild-type short peptide M08, could significantly promote the formation of branched CaPMNs compared to that of the untreated group (59% vs. 32%; Figure 1).

Next, we employed wild-type M08 aa sequence as a template and generated 17 mutations, including single-aa, multiple-aa, shorter-peptide, and longer-peptide mutants through both conservative and non-conservative aa substitutions. Using this zebrafish phenotypic screening platform, we evaluated the effect of various mutant peptides on the formation of branched CaPMNs compared to that of M08. As before, we employed ICV injection to deliver different mutant peptides individually into embryos, followed by an analysis of the morphological branch patterns of six CaPMNs, and three different levels of potency were obtained: (1) superior to that of M08, e.g., M039 with higher neuroprotective activity; (2) equal to that of M08, e.g., M029 and M036; and (3) inferior to that of M08, e.g., M027 and M044, which showed significantly reduced branched CaPMNs in zebrafish embryos, even less than the untreated control group. Therefore, we suggest that zebrafish can serve as an effective preliminary platform to differentiate the ability of peptide mutants to improve MN growth.

### 3.2. Zebrafish Phenotypic Screening Revealed That Mutant Short Peptide M039 Could Promote the Highest Formation of CaPMNs

Of all short-peptide mutants screened, including wild-type template (M08), results showed that single-aa-mutated M039, from which the original Glu at the 358th position of M08 was replaced by Arg, outperformed M08 in forming branched CaPMNs up to 75%, surpassing the parental M08 sequence (59%). In the next step, we employed our in vitro screening model, NSC34 neural cells, to determine the effect of M039 on axonal growth. Compared to M08, supplementary addition of M039 to the CM could more effectively promote NOMN derived from NSC34 cells, as well as substantially decrease p-Cofilin level, thus favoring neurite outgrowth through the novel pP38-T180/pMK2-T334/p-Limk1-S323/p-Cofilin-S3 signaling pathway, as reported by Lin et al. [20]. Finally, we employed ALS-like zebrafish embryos to further confirm that M039 could outperform M08 by more effectively rescuing the shortened axons and inferior locomotor function defects found in ALS-like zebrafish embryos. These results give further evidence that the zebrafish phenotypic screening platform is a reliable strategy for rapidly screening potential short peptides.

### 3.3. Arg Substitution for Glu at the 358th of Short Peptide M039 Might Strengthen Structural Stability and Interaction, Enhancing the Formation of Branched CaPMNs

We previously summarized our procedure for using aa of wild-type M08 as a template to generate 17 mutations, including single and multiple aa mutations through both conservative and non-conservative substitutions. We further defined conservative mutation as the substitution of an aa with similar physicochemical property, such as charge or polarity, and non-conservative substitution as a missense mutation where the change results in a chemically dissimilar aa such that the new, distinct property alters local structure or functional domains. Here, we provide additional detail to expand and clarify these steps.

The C-terminal region, consisting of the 325th to 417th aa residues of ligand Pgk1, has been shown to interact with the 404th to 431st aa residues of receptor Eno2, thereby promoting NOMN [21]. Lee et al. [22] demonstrated that NOMN was retarded in cells overexpressing Eno2-D419S, -E420K and -M22K mutants. Specifically, they showed that two negatively charged aa residues, D419 and E420, of Eno2 most likely interact with the 325th–417th domain of Pgk1, which is a positively charged segment that forms an α-helix secondary structure. E358 is one of the surface-exposed aa residues of ligand Pgk1 near Eno2 and has been structurally predicted to form an α-helix that interacts with the counterpart of D419 or E420 of receptor Eno2 within the Pgk1-Eno2 interface [22]. Given the opposing surface charges, we hypothesized that substituting Glu with Arg at position 358 in M039 might improve Pgk1-Eno2 interaction. Indeed, previous studies highlighted that single aa substitution could significantly impact protein stability and function. For example, Pedone et al. [27] reported that substituting Glu with Arg in *E. coli* thioredoxin could generate an additional hydrogen bond, thus improving thermal stability. Since Arg is a highly polar and strongly basic aa, its guanidinium group can form strong electrostatic attractions with negatively charged regions [28], thereby strengthening structural stability and binding affinity. Consequently, Arg substitution for Glu at the 358th position of M039 appeared to alter the local three-dimensional shape owing to its long, positively charged side chain and non-covalent interactions.

In sum, the electrostatics of these interactions may improve the interface between short peptide M039 and receptor Eno2 [29], leading to increased stability and functional efficiency.

In the future, we would incorporate molecular docking and molecular dynamics (MD) simulations to structurally confirm how the Arg substitution influences the binding geometry, geometric complementarity, and energetic compatibility, thus validating the predicted mechanism for enhanced CaPMNs formation. Additionally, it would be worthwhile to investigate why charge-reversal changes at the other sites (346, 350, 353, 357) do not enhance charge attraction or increase functional potency compared to M08.

### 3.4. Mutant Peptides M027, M029, and M044 Selected from Zebrafish Screening Demonstrated Loss of Function Toward the Formation of Branched CaPMNs

In addition to the positive effect of M039, as described above, the zebrafish phenotypic screening platform could also differentiate mutant peptides, including M027 and M044, that showed a significant loss in the ability to promote the formation of branched CaPMNs in zebrafish embryos, even lower than mutants tested in the untreated control group.

M027 is a two-aa-substitution mutant in which Lys at position 353 (K353) is replaced by Arg (K353R) and combined with A354P. To explain the adverse effect caused by M027, we speculate that the double mutant K353R + A354P of M027 may interfere with the binding interface of Pgk1-Eno2 and hinder interaction because of increased charge and polarity (Table 1). It is also possible that local secondary structure of this key segment might be destabilized since the cyclic structure of proline, the limiting backbone of rotation, in turn, decreases flexibility [30,31]. Therefore, K353R + A354P of M027 leads to a substantial decline in the formation of branched CaPMNs down to 29%, even lower than control. Notably, M027 was designed by combining a single-aa mutation of M09 (A354P) and a single-aa mutation of M10 (K353R). Interestingly, supplementation of either M09 or M10 alone could maintain, or even improve, the formation of CaPMNs activity compared to that of M08. However, the combination of these two mutations together in M027 exhibited a negative effect, suggesting that the two aa, K353 and A354, may be tightly linked in the structural core of Pgk1-Eno2 interaction, such that disrupting these two linked aa simultaneously would abolish the functional activity of M027. In fact, multiple mutations might produce an antagonistic effect on three-dimensional conformation. This underscores the existence of nonlinear interactions (epistasis) in multi-mutant scenarios, rather than purely additive effects.

Another example is M029 which is mutated at three amino acids (K353R, E357R, and D358Q), increasing local positive charge (Table 1). Again, in theory, these substitutions of ligand short peptide should increase electrostatic or hydrogen-bond interactions with the negatively charged binding surface of receptor Eno2. However, results showed that the ability of M029 to form branched CaPMNs remained similar to that of wild-type M08, e.g., 60 vs. 59%, respectively (Figure 1). These results suggest that future studies should use molecular docking and molecular dynamics simulations to determine whether this triple mutation simply changes the accessible orientation of the peptide without significantly enhancing geometric complementarity and energetic compatibility, as described by Sheinerman et al. [32], Baker et al. [33] and Eberhardt et al. [34].

In the future, structural analysis and potentially utilizing computational modeling are needed to confirm the hypothesis that the combination K353R + A354P destabilizes the key interaction segment, rather than simply having an additive effect.

### 3.5. Effects of Charge-Reversal Mutation and Multiple Mutations of Short Peptides on the Improvement of Branched CaPMNs Formation

We studied charge-reversal in four mutants: M035 (E346R), M036 (R350D), M037 (K353D) and M038 (D357R). Results showed that the percentage of CaPMNs formation was 52.13 ± 5.79%, 56.47 ± 1.30%, 69.92 ± 8.99% and 54.83% for M035, M036, M037 and M038, respectively (Figure 1). Since none of these results significantly deviated from that of M08 (59%), it can be concluded that charge-reversal changes at these particular sites of short peptides would not increase the charge attraction between Pgk1 and Eno2. In this study, we did find that only one charge-reversal mutant, M039 (E358R), could generate a significant functional improvement of branched CaPMNs formation compared to M08, suggesting that the 358th aa residue is a promising target for functional improvement. Thus, it is reasonable to consider that the positively charged 358th aa may be a hotspot for positive optimization [35,36].

Furthermore, we studied three peptide mutants to understand the cumulative effect of multiple aa substitutions: M041 containing four mutations (A347P, R350D, D357R, and E358R), M042 containing five mutations (mutated K353R was added into M041), and M043 containing six mutations (mutations K353D and A354P were added into M041). As shown by the results, these three mutated short peptides could drive the formation of branched CaPMNs by 55, 44 and 50%, respectively, none of which were greater than that of M08 (59%), suggesting that the overall structure tolerates simultaneous charge-reversals and rigidity modifications and that excessive accumulation of positive charges may disrupt optimal electrostatic balance or binding geometry as described by Sheinerman et al. [32]. Thus, based on these results, we hypothesize that the compensatory effects among multiple aa mutations may offset the positive effect of single-mutated short peptide.

Finally, we found that M044 with 12 aa has sequence identity equal to that of M029, except for the absence of two aa at each end (Table 1), leading to a reduction to 38.81% in forming branched CaPMNs, while the branched CaPMN-forming ability of M029 was otherwise similar to that of M08, e.g., 60 vs. 59%, respectively. These results indicate that peptides with a length as short as 12 aa would fail to improve branched CaPMNs formation. On the other hand, we found that the percentage of branched CaPMNs formed by M040, which is composed of 21 aa in which five more aa (KATSR) are added at the C-terminus of M08 (Table 1), was distinguishable from that of M08, e.g., 69 vs. 59%, suggesting that five extra aa residues at the C-terminal from 360th to 365th might favor a more stable three-dimensional structure and electrostatic distribution microenvironment for Pgk1-Eno2 interaction, resulting in improving the formation of branched CaPMNs. Future studies using molecular docking, molecular dynamics, and electrostatic analyses are needed to confirm how this mutation influences binding geometry and energy landscapes. Future efforts should focus on determining the minimal sequence length required for maintaining the three-dimensional structure and optimal electrostatic distribution for Pgk1-Eno2 interaction, and confirming the specific contribution of the five extra C-terminal residues in M040 (KATSR).

### 3.6. Zebrafish-First Strategy Followed by Cell-Based Mechanistic Validation

In recent years, multiple studies have adopted a “zebrafish-first, back-to-cells” strategy whereby phenotype validation in zebrafish precedes mechanistic exploration in cells or tissues. For example, Clemizole was initially identified in an SCN1A-mutant zebrafish epilepsy model as an anti-seizure compound and was later validated in vitro to act via the 5-HT_2_B receptor [37]. Leflunomide was found to inhibit neural crest cell development in zebrafish chemical-genetic screening and was subsequently confirmed as a dihydroorotate dehydrogenase inhibitor in mammalian cells and melanoma models [38]. ORC-13661 was first demonstrated to protect zebrafish lateral line hair cells from ototoxicity and later validated in mouse outer hair cells and in vivo models to block a mechano-transduction channel [39]. Likewise, dmPGE_2_ was discovered in zebrafish to increase hematopoietic stem cell numbers and later confirmed in mouse and human explants to regulate the Wnt pathway, promoting the expansion of hematopoietic stem cells [40]. These examples highlight the translational value of zebrafish as a rapid in vivo screening platform that can pinpoint candidate molecules and guide mechanistic validation in higher systems.

Our study showed that Pgk1-derived short peptides have sequence–function relationships wherein a single aa change may greatly affect peptide activity. This highlights the importance of designing sequences that target protein–protein interaction interfaces. A zebrafish phenotypic screening platform allows for quick initial screening, while mammalian models are more time-consuming and resource-intensive. Zebrafish also can provide high-throughput pharmacological and behavioral studies in a short period, complementing cell models that only offer molecular and local mechanistic insights [3,9]. By combining conservative and non-conservative substitutions, as described in detail above, it becomes possible to systematically identify critical residues within Pgk1-derived peptides. This dual approach—biophysical adjustment through aa substitution and in vivo functional testing—speeds up candidate discovery and avoids the inefficiencies of traditional random drug development [4,5,41]. Reciprocal validation using zebrafish and cell systems not only strengthens experimental reliability but also highlights zebrafish as a rapid, reliable, convenient and translational platform for early drug discovery in neurodegenerative diseases.

### 3.7. Zebrafish Platform Applied for Screening Potential Peptide Drugs

Only a few studies have explored zebrafish as a model for peptide drug screening. For example, in a Pentylenetetrazole (PTZ, a GABA-A receptor antagonist commonly used to induce seizures) zebrafish model, investigators tested the tripeptide p-BTX-I (derived from snake venom β-bungarotoxin) and the connexin-43-derived peptide CX2 (from gap junction protein connexin-43). Both peptides were shown to modulate seizure frequency and improve swimming behavior [39,42]. The study also examined peptide biodistribution in vivo, providing a clear demonstration of zebrafish as a model for peptide validation. Additionally, Ramachandran and Rajagopal [43] discussed zebrafish as a platform for pharmacological screening of marine peptides and toxins with both anticancer and cardioprotective effects; similarly, Shi et al. [44] highlighted the utility of zebrafish in evaluating bioactive natural products and peptides. However, these studies mostly remain at the level of in vivo phenotypic validation without integrating cellular and molecular mechanistic analyses. In contrast, our work advances this framework by prioritizing a zebrafish-first, back-to-cells strategy. We initially used zebrafish embryos to rapidly screen for bioactive short peptides and then test the top candidates in ALS-like zebrafish embryos to confirm their ability to rescue axonal shortening and locomotor defects. If validated, we will further advance to mechanistic cell models cultured in ALS-like conditions to examine p-Cofilin level and NOMN as final readouts. This approach identifies mutants with the strongest neuroprotective potential. The consistency of our results strengthens the reliability of our findings. Collectively, our study demonstrates the application of zebrafish in peptide drug development, providing a new drug discovery paradigm, i.e., a sequence design–based screening strategy integrated with zebrafish validation. This framework not only accelerates the discovery of candidate peptides but also ensures mechanistic confirmation, offering a translationally valuable workflow for advancing peptide drugs. More importantly, after the designed mutated peptides subsequently undergo a series of in vivo and in vitro bioactivity assays, the results should provide a comprehensive elucidation of the contribution of critical aa residue(s) in Pgk1-derived short peptides to their neuroprotective functions.

## 4. Materials and Methods

### 4.1. Pgk1-Derived Peptide and Its Mutants Designed for This Study

Short peptide M08, a truncated form of the 345th to 360th aa segment of full-length Pgk1, served as a template. We employed both conservative and non-conservative site-directed mutagenesis to design 17 M08-derived mutants (Table 1). Conservative mutation aims to substitute an aa with similar physicochemical property (e.g., charge, polarity, or molecular size) to maintain the overall structural stability of peptide, while assessing the effect of side-chain modifications on functional activity. Based on the M08 sequence, we altered the 353rd residue from lysine (K) to arginine (R) to increase its positive charge, generating peptide M010 with which to explore the contribution of altered charge intensity to neurotrophic function. Meanwhile, a non-conservative mutation introduces a missense substitution where the change results in a chemically dissimilar aa such that the new, distinct property alters local structure or functional domains, thereby identifying the necessity of specific residues for peptide activity. From the perspectives of charge and polarity, we designed several peptides, including M09, M022, and M031-M039, by systematically altering site-specific physicochemical properties. Furthermore, to evaluate the synergistic effects of multiple mutations, we combined several substitutions to generate complex mutants, such as M027-M030 and M040-M044, in which an extra five residues were added in M040, while two aa residues were deleted from both N- and C-termini in M044.

### 4.2. Peptide Synthesis and Preparation

Peptides were custom-synthesized by Mission Biotech (Taipei, Taiwan) with specifications that included defined aa length, desired quantity (mg), and a minimum purity of >90%. Lyophilized peptides were stored at −80 °C and protected from light. Peptide reconstitution and preparation followed Merck’s Synthetic Peptide Handling and Storage Protocol. Peptides were dissolved in PBS.

### 4.3. Zebrafish Husbandry and Transgenic Line

The maintenance, breeding, and developmental stage determinants of zebrafish followed the protocols described by Westerfield [45]. The *Tg(mnx1:GFP)* transgenic line [46], which expresses green fluorescent protein in MN, was purchased from Zebrafish International Resource Center, University of Oregon (Eugene, OR, USA).

### 4.4. Formation of Branched CaPMNs in Zebrafish Embryos

We followed the protocol described by Lee et al. [22] with some modifications. Briefly, after the short peptide was delivered into *Tg(mnx1:GFP)* embryos at 20–22 hpf through ICV injection at a dosage of 9.2 ng, we counted the number of embryos with branched CaPMNs formation between the 10th and 16th somites in the left trunk of *Tg(mnx1:GFP)* embryos at 30 hpf under inverted fluorescence stereo microscopy (Olympus IX71, 200× magnification, objective: 40×, Olympus, Tokyo, Japan). Each experiment was performed with 15–35 embryos, and the average datum was represented. Some target experimental groups were performed at least three independent trials. To standardize data presentation, the mean value obtained from each independent experiment was treated as one data point, and the final results were expressed as the grand mean of all experimental trials (mean ± SEM). The sample size (“*n*”) indicated the total number of embryos analyzed across all trials. Because the zebrafish assay served as a tiered and primary screening platform designed to efficiently identify peptide candidates while reducing unnecessary experimental repetition, peptides that showed no apparent difference from the benchmark M08 during the initial evaluation were examined in only 1–2 independent experiments. In contrast, peptides that displayed a clear trend of being better or worse than M08 were subjected to three or more independent repeats to ensure consistency and reliability.

### 4.5. Knockdown of C9ORF72 and Axonal Length in Zebrafish Embryos

To obtain ALS-like zebrafish, we microinjected antisense MO specific for *C9ORF72* (TTGTAACATCCAT-CTGCTGCTGCAT) into embryos at the one-cell stage in a concentration of 1 ng. Embryos were collected and fixed at 30 hpf and then analyzed using fluorescence stereo microscopy (Leica M205 FCA, Wetzlar, Germany) under a 10× objective. The length of each axon between the 11th and 15th somites on the left trunk was measured (in μm) using ImageJ version 1.54g. Datum of each embryo was averaged from these five axons, and the datum of each group was represented by four embryos for each experiment. The total data of each group from three independent experiments were all presented, and the mean value was averaged from 12 embryos.

### 4.6. Tracking the Swimming Trajectory of Zebrafish Larvae

Zebrafish embryos were placed individually in 3 cm diameter agar-coated dishes containing 3 mL of water. Tail touch-evoked escape response [26] was performed at 72 hpf whereby the tail of each larva was gently stimulated with a fishing line to induce short-range swimming. When the larva temporarily stopped, a second touch was consecutively performed. Each larva received a total of three stimulations. Swimming trajectory and total moving distance (in cm) were recorded and analyzed using EthoVision XT 17.5 software. Datum of each embryo was averaged from three trials, and datum of each group was represented by 10 embryos for each experiment. The total data of each group from three independent experiments were all presented, and the mean value was averaged from 30 embryos.

### 4.7. Culture Conditions of NSC34 Neural Cells and Sol8 Myoblasts

NSC34 neural cells provided by Dr. Cashman [47], SOD1-G93A ALS-like NSC34 cells [48], Sol8-vector and Sol8-NogoA myoblasts [20] were cultured in Dulbecco’s Modified Eagle Medium (DMEM; Gibco, Waltham, MA, USA) supplemented with 10% fetal bovine serum (FBS; Gibco), 100 U/mL penicillin, and 0.1 mg/mL streptomycin (Biowest, Nuaillé, France) at 37 °C in a humidified atmosphere containing 5% CO_2_. For Sol8-vector and Sol8-NogoA cells, 4 μg/mL puromycin was added during passage for stable cell lines, and 1 μg/mL doxycycline was applied to induce NogoA overexpression.

### 4.8. NOMN Derived from NSC34 Cells Cultured in CM

Procedures followed the protocol described by Lee et al. [22] with minor modifications. Briefly, Sol8-vector or Sol8-NogoA cells were seeded at a density of 3 × 10^5^ cells per 10 cm dish and cultured in DMEM high glucose medium containing 1.5% FBS to generate CM. NSC34 cells were seeded at a density of 3 × 10^3^ cells per well on cell culture slides with two wells and cultured with CM supplemented with Pgk1-derived peptide in a concentration of 99 ng/mL. The control group was treated with PBS for four days. Cells were immunostained with α-tubulin (Sigma, RRID: AB_477579; 1:5000) and anti-mouse Cy3 secondary antibody (Sigma, RRID: AB_11213281; 1:250), followed by counterstaining with DAPI (Sigma, CAS:28718-90-3). For each experiment, ten fields of each sample were randomly selected and observed with a Leica TCS SP8 confocal microscope under 20× objective (Leica Microsystems, Wetzlar, Germany). Total NOMN was quantified using the MetaMorph neurite outgrowth analysis module. For each trial, the neurite length of NOMN for each group was averaged from 100 cells with relatively longer neurites. The final data was averaged from three independent trials. The percentage of neurite-bearing cells with neurites over 100 μm in length among the 100 examined cells in each group was also quantified. The final data of each experimental group was averaged from its three independent trials.

### 4.9. Protein Level of p-Cofilin Expressed in ALS-like NSC34 Neural Cells

SOD1-G93A ALS-like NSC34 neural cells were seeded at a density of 1 × 10^5^ cells per well in a 6-well plate and cultured in DMEM with 10% FBS overnight. Then, cells were cultured in DMEM containing 1% FBS and supplemented with 99 ng/mL short peptide. After 24 h, total cellular proteins were extracted.

### 4.10. Protein Extraction from Cells

Total protein extraction was performed according to Lin et al. [49]. Briefly, cells were washed with PBS, harvested by scraping, and collected into microcentrifuge tubes, followed by centrifugation at 3000 rpm for 5 min. The pellet was lysed in whole cell extract (WCE) buffer containing cOmplete™ Protease Inhibitor Cocktail tablets (EDTA-free; Sigma-Aldrich, St. Louis, MO, USA) and PhosSTOP™ (Sigma-Aldrich). Samples were vortexed for 5 s and incubated on ice for 10 min with additional vortexing every 5 min. Lysates were centrifuged at 13,000 rpm for 15 min at 4 °C, and the supernatant was saved. After protein concentrations were determined by the Bradford assay, samples were ready for Western blot analysis.

### 4.11. Western Blot Analysis

Western blot analysis followed the protocol described by Lee et al. [22]. Briefly, proteins were separated on 12% polyacrylamide gels and transferred to PVDF membranes. Membranes were blocked with 5% BSA in Tris Buffered Saline with Tween 20 (TBST) for 1 h at room temperature, followed by overnight incubation with primary antibody under gentle shaking at 4 °C. After washing, membranes were incubated with secondary antibody for 1 h at room temperature. Protein bands were visualized using an Invitrogen iBright™ CLC750 imaging system with ECL substrate (Immobilon^®^ Western Chemiluminescent HRP substrate, Millipore, Taipei, Taiwan). Densitometric quantification of protein expression was performed using ImageJ software. α-tubulin served as the internal loading control.

### 4.12. Statistical Analysis

All experiments were independently repeated at least three times. Data are expressed as mean ± SEM. Statistical analysis was performed using one-way analysis of variance (ANOVA), followed by Tukey’s post hoc multiple comparison test. A value of *p* < 0.05 was considered statistically significant at the level of * *p* < 0.05, ** *p* < 0.01, and *** *p* < 0.001.

## 5. Conclusions

In this study, we established a multilevel screening platform that integrates zebrafish and cellular models to evaluate the neuroprotective potential of Pgk1-derived short peptides systematically. Among 17 engineered mutants, M039, a single aa-substitution Glu358-to-Arg358, exhibited the most pronounced improvement of NOMN in NSC34 neural cells. This finding was further supported by the significantly reduced p-Cofilin level in ALS-like NSC34 neural cells treated with short peptide M039. Moreover, CaPMNs branching and partial rescue of axonal growth and motor function were more much significantly improved in ALS-like zebrafish embryos treated with M039. Our results demonstrated that the zebrafish-first strategy enables rapid identification of functional peptide variants, while subsequent cellular assays confirm molecular mechanisms. This sequence-guided, phenotype-driven framework offers a translationally valuable pipeline for discovering neuroprotective peptides and advancing them toward therapeutic development for neurodegenerative disorders such as ALS.

## Figures and Tables

**Table 1:** Amino acid sequences of Pgk1-derived peptides containing site-specific mutations and extra additions or deletions of amino acids.

Peptide(s)	Sequence of Amino Acids from 345th to 360th of Pgk1																					Mutation Type	
																						Charge	Polarity
M08	W	E	A	F	A	R	G	T	K	A	L	M	D	E	V	V							
M09	.	.	.	.	.	.	.	.	.	P	.	.	.	.	.	.						#	+
M010	.	.	.	.	.	.	.	.	R	.	.	.	.	.	.	.						↑	*
M022	.	.	.	.	.	.	.	.	.	.	.	.	R	Q	.	.						↑	*
M027	.	.	.	.	.	.	.	.	R	P	.	.	.	.	.	.						↑	+
M028	.	.	.	.	.	.	.	.	.	P	.	.	R	Q	.	.						↑	+
M029	.	.	.	.	.	.	.	.	R	.	.	.	R	Q	.	.						↑	*
M030	.	.	.	.	.	.	.	.	R	P	.	.	R	Q	.	.						↑	*
M035	.	R	.	.	.	.	.	.	.	.	.	.	.	.	.	.						↑	*
M036	.	.	.	.	.	D	.	.	.	.	.	.	.	.	.	.						↓	*
M037	.	.	.	.	.	.	.	.	D	.	.	.	.	.	.	.						↓	*
M038	.	.	.	.	.	.	.	.	.	.	.	.	R	.	.	.						↑	*
M039	.	.	.	.	.	.	.	.	.	.	.	.	.	R	.	.						↑	*
M040	.	.	.	.	.	.	.	.	.	.	.	.	.	.	.	.	K	A	T	S	R	#	*
M041	.	.	P	.	.	D	.	.	.	.	.	.	R	R	.	.						↑	*
M042	.	.	P	.	.	D	.	.	R	.	.	.	R	R	.	.						↑	+
M043	.	.	P	.	.	D	.	.	D	P	.	.	R	R	.	.						↑	+
M044			.	.	.	.	.	.	R	.	.	.	R	Q								↑	*

Dot: Indicates the identical amino acid of the original sequence of wild type M08, while the mutated and extra added amino acid(s) were indicated. The charge of mutated peptide remained unchanged (#) or altered to become increase (↑) or decrease (↓) compared to that of M08. The polarity of mutated peptide remained unchanged (*) or increase (+) compared to that of M08.

## Data Availability

The data sets used and analyzed during the current study are available from the corresponding author upon reasonable request.
